# Genome wide association study in Swedish Labrador retrievers identifies genetic loci associated with hip dysplasia and body weight

**DOI:** 10.1038/s41598-024-56060-y

**Published:** 2024-03-13

**Authors:** Ida Nordang Kieler, Sofia Malm Persson, Ragnvi Hagman, Voichita D. Marinescu, Åke Hedhammar, Erling Strandberg, Kerstin Lindblad-Toh, Maja Louise Arendt

**Affiliations:** 1 Department of Veterinary Clinical Sciences, University of Copenhagen, Copenhagen, Denmark; 2 Department for Breeding and Health, Swedish Kennel Club, Stockholm, Sweden; 3 Department of Clinical Sciences, Swedish University of Agricultural Sciences, Uppsala, Sweden; 4 Department of Medical Biochemistry and Microbiology, Uppsala University, Uppsala, Sweden; 5 SciLifeLab, Uppsala, Sweden; 6 Department of Animal Breeding and Genetics, Swedish University of Agricultural Sciences, Uppsala, Sweden; 7 Broad Institute of MIT and Harvard, Cambridge, MA USA

**Keywords:** Animal breeding, Genetic association study

## Abstract

Genome wide association studies (GWAS) have been utilized to identify genetic risk loci associated with both simple and complex inherited disorders. Here, we performed a GWAS in Labrador retrievers to identify genetic loci associated with hip dysplasia and body weight. Hip dysplasia scores were available for 209 genotyped dogs. We identified a significantly associated locus for hip dysplasia on chromosome 24, with three equally associated SNPs (p = 4.3 × 10^–7^) in complete linkage disequilibrium located within *NDRG3*, a gene which in humans has been shown to be differentially expressed in osteoarthritic joint cartilage. Body weight, available for 85 female dogs, was used as phenotype for a second analysis. We identified two significantly associated loci on chromosome 10 (p = 4.5 × 10^–7^) and chromosome 31 (p = 2.5 × 10^–6^). The most associated SNPs within these loci were located within the introns of the *PRKCE* and *CADM2* genes, respectively. *PRKCE* has been shown to play a role in regulation of adipogenesis whilst *CADM2* has been associated with body weight in multiple human GWAS. In summary, we identified credible candidate loci explaining part of the genetic inheritance for hip dysplasia and body weight in Labrador retrievers with strong candidate genes in each locus previously implicated in the phenotypes investigated.

## Introduction

Canine genome wide association studies (GWAS) have been proven to be a powerful tool to identify genetic loci associated with both simple and complex inherited conditions in dogs^1–3^. Hip dysplasia (HD) occurs commonly in dogs and has a major impact on animal welfare and life expectancy for the affected dogs^4^. The disease is considered heritable with an estimated heritability of between 0.2 and 0.6 and a presumed polygenic etiology^5–7^. However, environmental factors such as excessive food intake and rapid weight gain in growing animals as well as certain types of physical activities can increase the risk of developing clinical disease^8,9^. Treatment for clinical disease includes pain management, physiotherapy, and surgical orthopedic procedures^10,11^. Due to the heritability of the disease, screening dogs within high-risk breeds, such as German shepherds and Labrador retrievers, prior to breeding has been a major focus from kennel clubs and dog breeders as this has been shown to reduce frequency of moderate and severe HD^12,13^. Screening for HD is done by radiographic examination of the hip joint with the legs extended and abducted whilst the animal is heavily sedated or anaesthetized to allow for relaxation of the joint. Radiographic images are subsequently graded based on a standardized radiographic scoring scheme. The procedure for radiographic examination and subsequent grading has been standardized within the Fédération Cynologique Internationale (FCI)^14^. It has been shown that there is a good correlation between the radiographic evaluation of hip conformation and the development of clinically detectable HD later in life. Hence, selected breeding based on HD screening can be expected to reduce the prevalence of HD over time^4,12,13^. However, due to the complexity and presumed polygenic inheritance of HD as well as environmental factors influencing the phenotypic development, radiographic screening of breeding stock is not a warranty for unaffected offspring. Hence, other predictive measures such as genetic testing combined with estimated breeding values (EBVs) would provide more robust tools for breeding strategies against HD^12^.

Multiple studies have investigated the genetic cause of HD in dogs by GWAS varying in the number of SNPs used, the breed of interest and the phenotypic HD classification^15–20^. Although there is overlap between risk loci identified between some studies, and some studies investigate previously identified risk alleles in independent populations, no studies so far have validated that genetic markers can be used as a complement or as an alternative to radiographic screening. Several published genetic studies have focused on Labrador retrievers, as this is a popular breed which has been shown to have a higher risk of developing HD than some other breeds^4,6,15,18,19,21,22^. However, a genetic test based on a previous GWAS study in Labrador retrievers failed to correlate with the HD score when tested in an independent Labrador retriever cohort^15,23^.

Body weight or body size are highly heritable but are complex traits as evidenced by studies both in humans and animals^24–26^. The Labrador retriever is a dog breed which is known to be food motivated and at risk of developing obesity^27,28^. Previously, a deletion affecting the *POMC* gene was found to be associated with body weight and food motivation in Labrador retrievers^29^. As obesity is rarely inherited as a monogenic trait, it is likely that additional genetic loci play a role for these traits in Labradors^30^.

The aim of this study was to perform GWAS analyses on Labrador retrievers to identify genetic risk loci for HD and body weight based on Swedish Kennel Club (SKK) registered phenotypic data. In addition, color phenotypes were included as examples of simple Mendelian inherited phenotypes, to show proof of the method used for correction of population structure and data inflation in this cohort.

## Results

### GWAS analysis using color as the dependent phenotype

Genotyping data and color phenotypes were available for 209 SKK registered Labrador retrievers consisting of 205 females and 4 males (supplementary file S1). As a proof of concept, we performed a GWAS analysis using color as the dependent variable. This was performed to confirm that known genetic loci could be identified using this dataset and to show that the linear mixed model was able to correct for population structure and data inflation. We evaluated the population structure within the genotyped dogs by drawing a multidimensional scaling (MDS) plot depicting the two first dimensions (C1 and C2) and reflecting the color phenotype for each individual dog (Supplementary Fig. S1). The MDS plot showed that there is some population structure within the genotyped dogs with an unequal distribution of the brown phenotype across the dimensions.

We performed two separate GWAS analyses using color as the dependent phenotype. The first analysis compared black versus yellow dogs. This analysis included 148 black and 38 yellow Labrador retrievers with 115,072 SNPs passing data filtering. A basic association test correctly identified the *MCR1* locus on chromosome 5 as the most associated locus (chr5: 62,870,500, p_Black/yellow_ = 1.4 × 10^–22^), however, the results were inflated with a lambda value of $\uplambda$=1.39, as seen on the QQ-plot (Supplementary Fig. S2a and b). A subsequent analysis, using a linear mixed model, corrected for the data inflation with a resulting lambda value of $\uplambda$ =0.96 and also correctly identified the *MCR1* locus (chr5:64,358,997, p_Black/yellow_ = 1.49 × 10^–28^), (Supplementary Fig. S2c and d).

The second analysis was performed comparing 148 black against 22 brown Labrador retrievers. A basic association test correctly identified the *TYRP1* locus as the most associated locus (chr11:33,578,561, p_black/brown_ = 1.8 × 10^–38^). However, the results were inflated with a lambda value of $\uplambda$ =3.06, as visualized on the QQ-plot (Supplementary Fig. S3a,b). Applying a linear mixed model resulted in a mild overcorrection of the inflation with a lambda value of $\uplambda$ = 0.90 with the most associated locus (chr11:33,578,561, p_black/brown_ = 7.0 × 10^–40^) still being identified as the *TYRP1* locus (Supplementary Fig. S3c,d).

The “chip heritability”, i.e. the percentage of the phenotypic variation, that could be explained by the genetic variation in the genotyping data (PVE), was calculated for each trait using GEMMA^31^. This resulted in PVE_black/yellow_ = 0.96 +/− 0.16(SE) and the PVE_black/brown_ = 0.93 +/− 0.12(SE), which is in alignment with expectations for the inheritance of these color traits^32^.

Having showed that the linear mixed model was able to correct for genomic inflation, whilst correctly identifying the color loci, we applied this analytical method to analyze our data with HD grading and body weight as the dependent variables.

### GWAS analysis using the numerical HD score as the dependent variable

**Figure 1:**
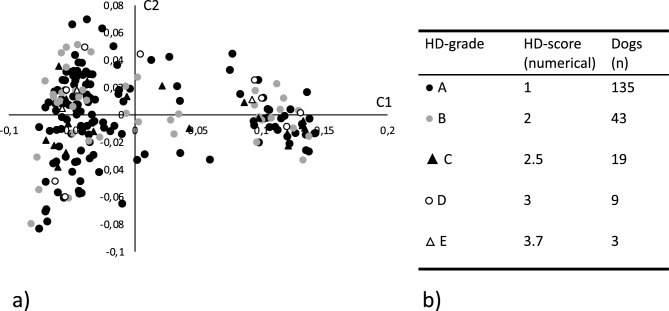
(**a**) Multidimensional scaling plot showing the genetic distance between the 209 genotyped Labrador retrievers in a two-dimensional space. Each datapoint represents an individual dog and the shape and color of the datapoint reflects the hip dysplasia (HD) score for the individual. Filled circle Represents individuals with grade A HD score (Normal hip score A). Grey circle represents individuals with grade B HD score (Normal hip score B). Filled triangle Represents individuals with grade C HD score (Mild hip dysplasia). Open circle Represents individuals with grade D HD score (Moderate hip dysplasia). Open triangle $\uprho$ represents individuals with grade E HD score (Severe hip dysplasia). (**b**) Summary of the actual and numerically translated HD score and the number of individuals in each category of the study population.

**Figure 2:**
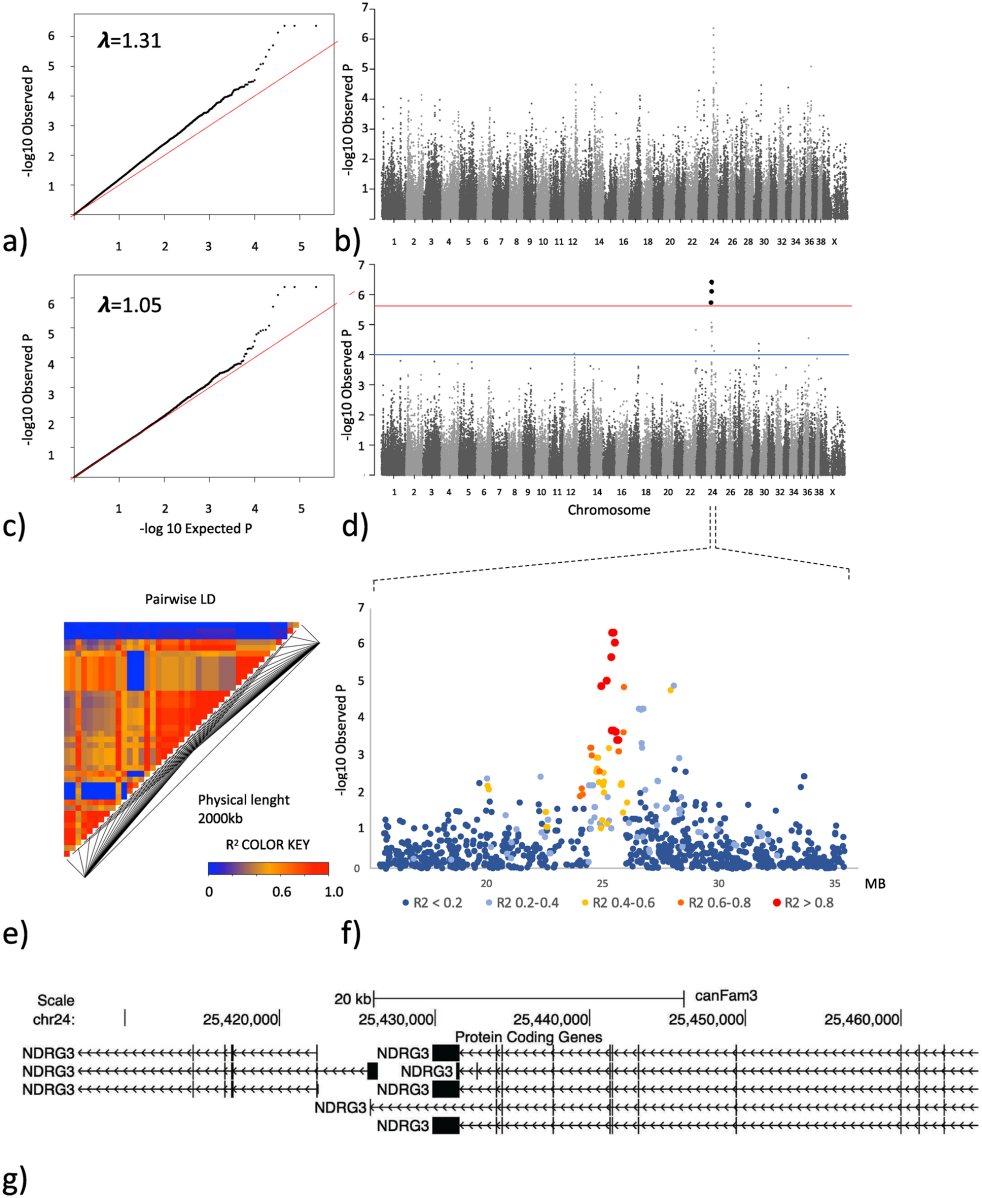
GWAS analysis based on hip dysplasia scores in 209 Labrador retrievers. (**a**) QQ-plot showing the expected versus the observed p-value from the basic association test. (**b**) Manhattan plot showing the genetic position across the 39 chromosomes versus the −log10 transformed p-value from the basic association test. (**c**) QQ-plot showing the expected versus the observed p-value from the linear mixed model association test. (**d**) Manhattan plot showing the genetic position across the 39 chromosomes versus the −log10 transformed p-value from the linear mixed model association test. The red line delineates the Bonferroni significance threshold. The blue line delineates a nominal significance threshold based on the deviation of the QQ-plot from the expected p-value. (**e**) LD plot reflecting the pairwise R^2^ between SNPs in a 2,000 kb window centered around the most associated SNP from association analysis. (**f**) Closer view of the associated locus on chromosome 24 reflecting the LD structure in relation to the most associated SNP (**g**) Annotated genome view of the area covering the three most associated SNPs on chromosome 24 which are in complete LD and are positioned within the coding and non-coding part of the *NDRG3* gene.

Genotyping data and matched numerically translated HD scores, as described in the materials and methods, were available for 209 SKK registered Labrador retrievers representing 205 females and 4 males. All individuals and 114,854 SNPs passed the quality control and filtering. An MDS plot showing the two first dimensions (C1 and C2), reflecting the numerical translated score of the included dogs, is shown in Fig. 1a together with a summary of the scores (Fig. 1b). As previously observed, the MDS plot shows some evidence of population structure that could affect the analytical process. A basic GWAS association test was performed in PLINK using the numerically translated HD value as the dependent continuous variable^33^. The analysis identified the strongest association to a locus on chromosome 24, with some degree of data inflation reflected in the QQ-plot and the lambda value $\uplambda$ =1.31 (Fig. 2a,b). A linear mixed model was applied correcting for the inflation, yielding a lambda value of $\uplambda$ =1.05 (Fig. 2c). A single significant locus was identified on chromosome 24, represented by five significantly associated SNPs, reaching the LD-corrected Bonferroni threshold (Fig. 2d, supplementary Table 1). The three strongest associated SNPs (chr24:25,406,999, chr24:25,417,878, chr24:25,465,028) were equally associated (p_HD_ = 4.3 × 10^–7^), in complete LD (R^2^ = 1.0), and are located within the *NDRG3* gene (Fig. 2e,f). One of these SNPs at chr24:25,465,028 is positioned in the splice donor position -3 of exon 4 in *NDRG*3. When evaluating the LD structure surrounding the most associated SNPs we found a 0.7 MB locus (chr24:24,945,587–25,656,879) in high LD (R^2^ > 0.8) with the most associated SNPs. This region encodes multiple candidate genes (*EPB41L1, AAR2, DLGAP4, MYL9, TGIF2, ENSCAFG00000029848, NDRG3, DSN1, SOGA1, C20orf118 and SAMHD1)* and the locus has previously been associated with HD in a GWAS performed in German shepherds and in a targeted study validating selected HD associated SNPs in Labrador retrievers^22,34^. A list of nominally significant loci was generated based on deviation from the expected p-value (supplementary file S2).

The percentage of phenotypic variability explained by the dataset, as calculated by GEMMA, was PVE_HD_ = 0.36 +/− 0.22(SE), which is in alignment with the previously reported heritability for HD in Labrador retrievers^7^. Body weight at the time of HD scoring was available for a subset of individuals n = 89. We examined if there was any difference in the body weight between the HD score groups however, we did not find any significant difference between groups p = 0.26 (Supplementary Fig. S4).

### Evaluation of EBV_HD_ in relation to the associated genotype on chromosome 24

Estimated breeding value is an indirect prediction of the genetic merit of a trait and can be used to predict the likelihood of an individual passing on a genetic trait to its offspring. The EBV for HD (EBV_HD_) was available for 205 of the 209 genotyped dogs. To validate the association between the identified risk genotype and HD status, we investigated if there was a difference in the EBV_HD_ between different genotype groups (Fig. 4a). The EBV_HD_ was compared between dogs that did not carry the risk allele and dogs that were either heterozygous or homozygous for the risk allele. As data was not normally distributed, as determined by a normality testing, Kruskal–Wallis and Dunn’s multiple comparison test was used to compare the EBV_HD_ between groups. We found that there was a significant difference between the mean EBV_HD_ in the groups (p = 0.003) (Table 1). When performing intergroup comparisons, we found that dogs that were homozygous for the risk allele had significantly lower (i.e. worse) EBV_HD_ than dogs that did not carry the risk allele (p = 0.0023) or dogs that were heterozygous for the risk allele (p = 0.023) (Fig. 4a).

**Figure 3:**
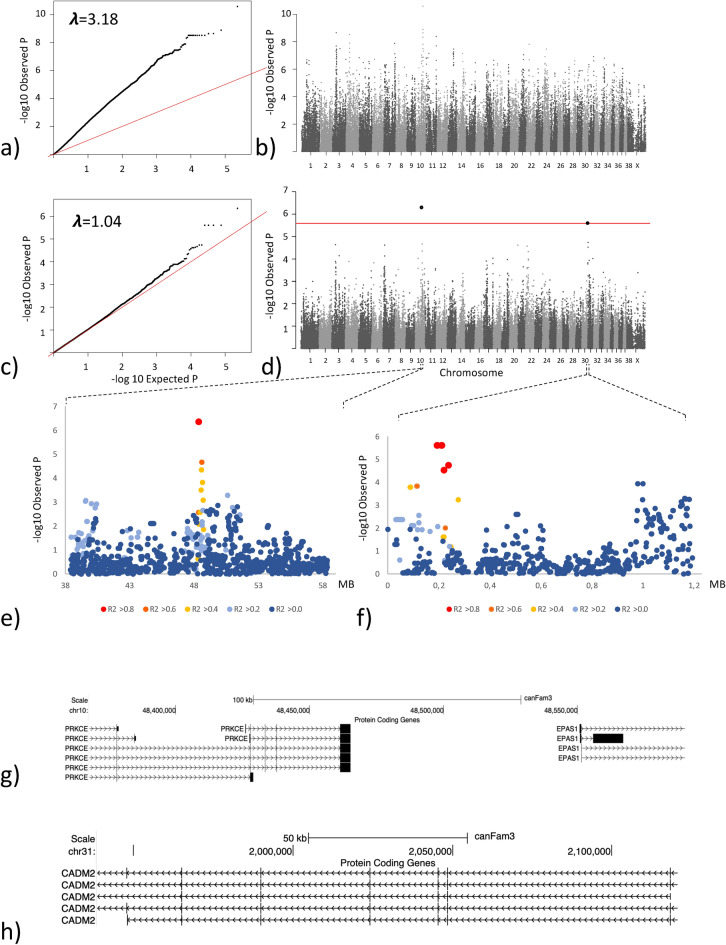
GWAS analysis based on the body weight of 85 female Labrador retrievers. (**a**) QQ-plot showing the expected versus the observed p-value from the basic association test. (**b**) Manhattan plot showing the genetic position across the 39 chromosomes versus the −log10 transformed p-value from the basic association test. (**c**) QQ-plot showing the expected versus the observed p-value from the linear mixed model association test. (**d**) Manhattan plot showing the genetic position across the 39 chromosomes versus the −log10 transformed p-value from the linear mixed model association test. The red line delineates the Bonferroni significance threshold. (**e**) Closer view of the associated locus on chromosome 10 reflecting the LD structure in relation to the most associated SNP (**f**) Closer view of the associated locus on chromosome 31 reflecting the LD structure in relation to the most associated SNP (**g**) Annotated genome view of the locus on chromosome 10 delimited by the most associated SNP and the two SNPs in highest LD with the associated SNP. (**h**) Annotated genome view of the area covered by the four most associated SNPs on chromosome 31 which are equally associated and in complete LD.

**Table 1:** Summary of the statistics from the data analysis shown in Fig. 4 a-c). P-values for the Kruskal–Wallis test (EBV_HD_) and the one-way ANOVA (body weight) are shown.

Genotype chr24:25406999	A/A	A/G	G/G	P-value
Mean EBV_HD_	99.1	97.7	86.0	**0.003**
Number of individuals	147	48	10	

**Genotype chr10:48367568**	C/C	C/T	T/T	P-value
Mean weight in Kg	21.8	27.8	29.5	**3.18 × 10**^**–12**^
Number of individuals	14	27	44	

**Genotype chr31:1938609**	C/C	C/T	T/T	P-value
Mean weight in Kg	26.6	31.6	33.5*	**2.45 × 10**^**–6**^
Number of individuals	67	17	1

**Figure 4:**
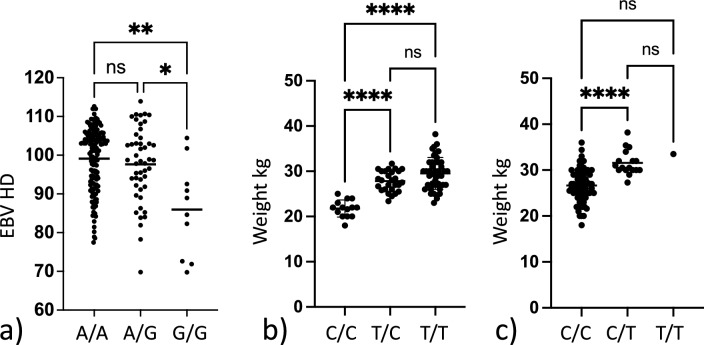
Box plots showing differences in phenotypic variables dependent on genotype. (**a**) Box plot showing the difference in mean estimated breeding value for HD (EBV_HD_) between dogs homozygous non risk (A/A) heterozygous risk (A/G) and homozygous risk (G/G) for the SNP chr24:25,406,999 associated with hip dysplasia. Significant difference is observed within the dataset. Group-wise comparison shows that homozygous risk individuals have a significantly lower (i.e. worse) EBV_HD_ than other dogs. (**b**) Box plot showing the difference in mean body weight between dogs homozygous non risk (C/C) heterozygous risk (C/T) and homozygous risk (T/T) for the SNP chr10:48,367,568 associated with increased body weight. Significant difference is observed within the dataset. With group wise comparison homozygous non risk individuals have a significantly lower body weight than the other groups. (**c**) Box plot showing the difference in mean body weight between dogs being homozygous non risk (C/C) heterozygous risk (C/T) and homozygous risk (T/T) for the SNP chr31:1,938,609 associated with increased body weight. Significant difference is observed within the dataset. With group wise comparison homozygous non risk individuals have a lower body weight than heterozygous risk carriers. Not significant (ns) = p-value > 0.05, * = p-value ≤ 0.05, ** = p-value ≤ 0.01, *** = p-value ≤ 0.001, **** = p-value ≤ 0.0001.

### GWAS analysis using body weight as the dependent phenotype

Body weight measured at the time of HD scoring was available for 89 dogs, 85 females and 4 males. As body weight differs significantly between males and females, we excluded the 4 males from the dataset to avoid an imbalanced sex bias^24^. To evaluate the effect of age on body weight, we performed a simple linear regression test comparing age and body weight. We did not find any correlation between increasing age and body weight (R^2^ = 0.0003 and p = 0.87 in the female dogs) (supplementary Fig. S5). We evaluated the population structure within the genotyped dogs by drawing an MDS plot depicting the two first dimensions (C1 and C2) and depicting dogs by weight groups. As previously seen the population structure is still observed in this smaller dataset (Supplementary Fig. S6). It is also noted that the weight categories are not equally distributed across the plot. GWAS analysis was performed using body weight as a continuous variable. After filtering and quality control, data from 85 dogs and 113,822 SNPs were included in the analysis. A basic association test was used to identify a locus on chromosome 10 as the most associated locus. However, the results were highly inflated with a lambda value of $\uplambda$ =3.18 (Fig. 3a,b). A second analysis using a linear mixed model, markedly reduced the inflation and resulted in a lambda value of $\uplambda$ =1.04. Two loci, on chr10:48,367,568, p_BW_ = 4.5 × 10^–7^ and on chr31:1,938,609, p_BW_ = 2.5 × 10^–6^, were identified with SNPs reaching the LD corrected Bonferroni threshold (Fig. 3c,d). The most associated SNP on chromosome 10 is located within an intron of the *PRKCE* gene, a gene previously linked to adipogenesis and body weight^35,36^. The locus on chromosome 10 is a narrow locus (Fig. 3e). The SNPs in highest LD with the locus defining SNP (R^2^ > 0.6) defines a 220 kb region containing the *PRKCE* and *EPAS1* genes (Fig. 3g). The locus on chromosome 31 included four equally associated SNPs in complete LD (R^2^ = 1.0) covering a 180 kb region spanning the *CADM2* gene (Fig. 3f,h), which is a gene that has been linked to obesity in multiple human studies looking at body composition in both children and adults^37–39^. In the NHGRI-EBI GWAS catalogue, BMI is listed as the most common trait associated with *CADM2*^40^. Evaluation of the LD structure surrounding the SNP marking the locus, showed a 450 kb locus in high LD (R^2^ > 0.8) with *CADM2* being the only gene located in the region.

The percentage of phenotypic variability explained by the dataset in relation to body weight, as calculated by GEMMA, was PVE_WEIGHT_ = 0.94 + /- 0.31(SE).

### Relationship between body weight and genotype for the two associated loci

**Figure 5:**
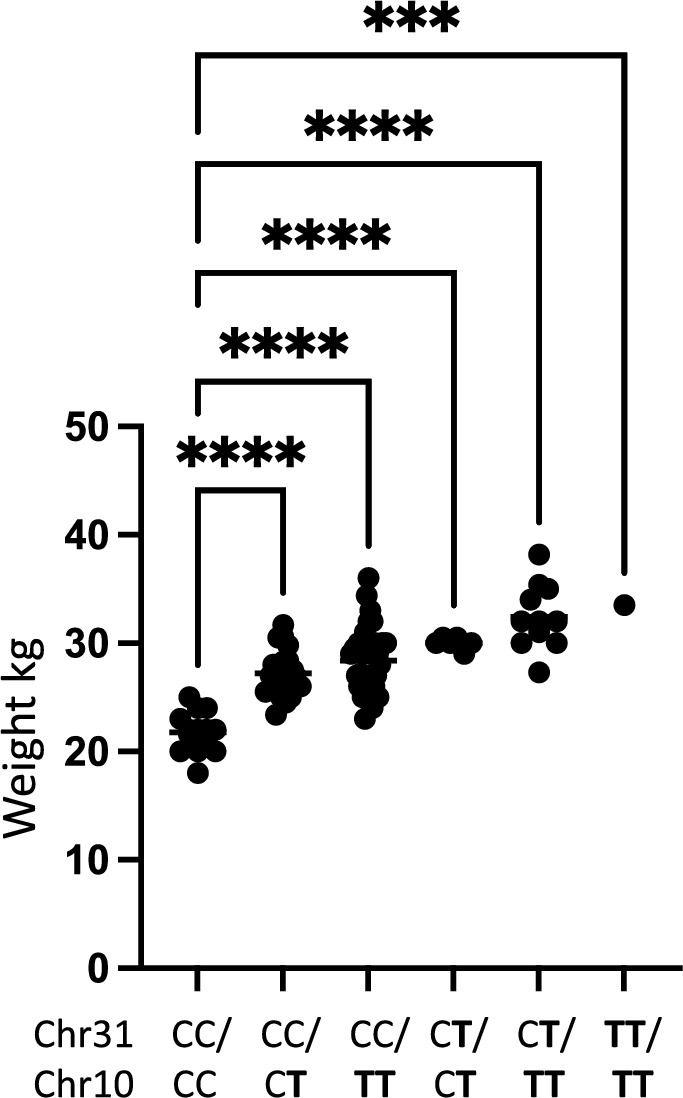
Boxplot illustrating the difference in weight between different groups. Labels indicate the genotype for the dogs in each group as reflected in Table 2 with T being the risk allele for each locus.

To evaluate the effect of genotype on weight we performed a one-way ANOVA analysis evaluating the difference between body weight in relation to the genotype for the two associated loci. We found that there was a significant difference in mean weight between the genotype groups representing the two risk loci on chromosome 10 p_BW10_ = 3.2 × 10^–12^ and chromosome 31 p_BW31_ = 2.4 × 10^–6^ (Table 1). Further, there was a significant difference in weight between dogs not carrying the risk allele on chromosome 10 and dogs being either heterozygous p_CC/CT_ = 4.4 × 10^–8^ or homozygous p_CC/TT_ = 6.7 × 10^–11^ for the risk allele. A significant difference between the weight of dogs not carrying the risk allele and dogs being heterozygous for the risk allele on chromosome 31 p_CC/CT_ = 3.5 × 10^–6^, was also found. However, only one dog was homozygous for the risk allele on chromosome 31 and hence no further meaningful statistical testing could be performed for homozygosity of this risk allele. Finally, a combined analysis was performed looking at the difference of body weight in dogs not carrying any risk alleles on chromosome 10 and chromosome 31 and dogs carrying different combinations of risk alleles for the two loci on chromosome 10 and chromosome 31 (Table 2 and Fig. 5). A statistically significant difference in body weight between dogs not carrying any risk alleles and dogs carrying any combination of risk alleles for the two associated loci on chromosome 10 and chromosome 31 was found.

**Table 2:** Summary of the comparison between individuals not carrying any risk alleles for increased body weight and individuals carrying different combinations of risk alleles representing the two associated loci on chromosome 10 and 31.

Genotype Chr 31/Chr10	N	Mean weight	P-value Dunnets multiple comparison to non-risk
C/C C/C	14	21.8	
C/C C/**T**	21	27.2	1.38 × 10^–7^
C/C **T**/**T**	32	28.4	3.18 × 10^–11^
C/**T** C/**T**	6	30.0	2.38 × 10^–8^
C/**T T**/**T**	11	32.5	2.40 × 10^–14^
**T**/**T T**/**T**	1	33.5	1.54 × 10^–4^ *

Risk allele shown in bold. P-values represent statistical comparison between the control group not carrying any risk alleles and groups carrying different combinations of risk alleles. *Only one sample was homozygous for both risk alleles and hence this should be taken into account when interpreting the data.

## Discussion

In this study a GWAS investigating the genetic association of HD and body weight in Labrador retrievers based on SKK registered phenotypic data, was performed. This identified a locus on chromosome 24 associated with HD scores in Labrador retrievers. Subsequently we evaluated if the EBVs_HD_ calculated by the SKK were different between groups either not carrying the risk allele or being hetero- or homozygotic for the risk allele. The EBV_HD_ was found to be lower (i.e. worse) in individuals carrying the risk allele, with the most significant effect observed in the group of individuals homozygous for the risk allele compared to the heterozygotes and non-carriers. The identified risk locus contains three equally associated SNPs in complete LD located within the *NDGR3* gene. *NDGR3* is a gene which has been shown to be strongly expressed in osteoarthritic joint cartilage in humans and is proposed to play a role in the hypoxia mediated development and progression of osteoarthritis^41,42^. A locus adjacent to the *NDRG3* locus on chromosome 24 has previously also been shown to be associated with HD in dogs. Fels & Distl (2014) performed a GWAS on German shepherds comparing 96 controls (dogs graded as normal, A or B, at screening) with 96 cases representing HD grade C–E, based on the FCI protocol^34^. In that study, in which a smaller and less dense SNP panel with 127 k SNPs was used, the most associated locus identified in the study was found on chromosome 24 with the most significant SNP being located at chr24:25,973,438, in the vicinity of the locus identified in our current study. That study validated the risk locus by genotyping the SNP in a cohort of 843 German shepherds including 277 non-dysplastic dogs and showed that the risk allele frequency was higher in the dysplastic group with an odds ratio of 4.7 (95% CI 3.72–5.94). In a more recent study by Mikkola et al. from 2021, a panel of 46 SNPs including the chr24:25,973,438 SNP were evaluated in 1600 dogs from 10 different breeds^22^. The study showed that the risk allele chr24:25,973,438, was associated with an increased odds ratio (OR = 1.87, 95% CI 1.07–3.28) of having HD in Labrador retrievers^22^. Finally, a study using a whole genome sequencing GWAS approach with phenotypes based on breed disease frequency rather than individual phenotypes, identified a locus overlapping with the current locus on chromosome 24. The locus was shown to be associated with anterior cruciate ligament rupture, elbow dysplasia and hip dysplasia in different breeds, including Labrador retrievers^17^. Overlap between the loci identified in these studies and the current study has been summarized in supplementary Fig. S7. With these previous studies in mind, the identified locus in the current study most likely has implications for HD in Labrador retrievers, though further functional and epidemiological validation studies are warranted to confirm the risk association and validate putative functional variants.

In our study, two loci significantly associated with body weight in female Labrador retrievers were identified. Subsequent testing showed that there is a significantly higher body weight in the group of individuals carrying either one or two copies of the risk allele in either locus. This was also confirmed when looking at the two risk loci combined. The risk locus on chromosome 10 is a narrow locus with the most associated SNP located in the *PRKCE* gene. This gene was identified as a novel locus associated with body weight and BMI in a large-scale human study investigating anthropometric traits^36^. The associated SNPs on chromosome 31 are located within the *CADM2* gene. A knockout of the *CADM2* gene was shown to protect against obesity in mouse models and *CADM2* has been identified as a risk locus for increased BMI in multiple human studies^37,39,40,43^. Further, evaluation of genotype dependent differential expression of *CAMD2* in different brain regions, including hypothalamus, has been shown to correlate with genotype in identified human *CADM2* risk alleles, validating their effect on gene expression^44^. With the comparative evidence for the *CADM2* gene’s role in obesity, this is a strong candidate locus for weight regulation in Labrador retrievers, which should be explored further to potentially understand the genetic cause of obesity within this dog breed.

The PVE explained by the data was calculated for all traits in this study. The PVE differed between the investigated phenotypes. Though the PVE was within the expected range for the HD and color association, it was higher than expected for the weight association, reflecting that body weight is a highly heritable trait for which a large proportion of the variation can be explained by genetics. Taking the confidence intervals into account, the PVE is not far from the heritability described for body weight within different dog breeds^24^. As we only had body weight and not body condition score, part of the weight phenotype in our study likely reflects dog size rather than solely obesity, which can explain the high PVE for this trait.

There are certain strengths and limitations in this study. We used a conservative threshold for determining significance, by using the significance threshold established by Bonferroni correction based on the number of independent SNPs in the dataset. Other GWAS studies have used different thresholds such as nominal significance based on deviation of the observed p-value from the expected p-value based on the Q-Q plot^2^. Here we employed a conservative threshold increasing the risk of missing possible loci not meting the conservative cut-off. However, the SNPs marking additional putative loci have been summarized in Supplementary file 2. In the vicinity of these SNPs it is observed that there are many additional candidate genes for future exploration. A strength of the current study is the high-quality phenotypic data being registered systematically in the SKK database and being collected in a stringent manner. However, the current study population only consisted of 31 dogs with HD scores C or worse, and a larger dataset with more severely affected individuals could have increased the power of detection. Another limitation is the inclusion of only female dogs in the weight analysis. This was done to avoid a strong bias towards the X chromosome, which is commonly found when performing an analysis including individuals of both sexes unless the X chromosome is discarded from the analysis^45^. We used body weight and not body condition score, or another more accurate measure of obesity, in this study and hence we cannot exclude that the weight reflects general larger body size and not necessarily obesity. Nevertheless, the associated loci identified have previously been associated with body mass and body weight in humans and animals.

In conclusion, this study identified genetic loci significantly associated with HD and body weight in Swedish Labrador retrievers. The identified loci intersected promising candidate genes with known function related to the observed phenotypes. Further validation studies should be performed in the future to fully understand the functional consequences of the risk SNPs and to identify putative causal variants.

## Materials and methods

### Sample collection and phenotypes

Ethylenediaminetetraacetic acid (EDTA) preserved blood samples were collected from Labrador retrievers as part of two studies investigating the genetic predisposition to severe uterine infection (pyometra) and atopic dermatitis in Swedish Labrador retrievers. As most samples were collected as part of the pyometra study, the dataset has an overrepresentation of females. Phenotypic data consisting of color, weight, HD score and individual HD estimated breeding value for HD (EBV_HD_), was extracted from the SKK registry based on the individual dog’s registration number. The EBV_HD_ reflects the likelihood of the dog passing on HD to their offspring and are expressed in relation to the breed average which is set at 100. Values above 100 reflect better than breed average and a decreased propensity to pass on HD to offspring, whilst values below 100 reflect worse than breed average and an increased propensity to pass on HD to offspring. HD scores based on the official protocol of the FCI with grades A–E were available for 204 dogs. Scores A and B represent normal (non-dysplastic) hip joints, C mild HD, D moderate HD and E severe HD. The scores were translated into a numerical value based on the scale presented by Malm et al.^46^. HD scores were available for 204 dogs based on the current FCI approved system. However, for a subset of five individuals the hips were scored based on an older Swedish scoring system which was translated into the FCI approved system^12,46^.

### DNA extraction

Genomic DNA was extracted from EDTA blood using the QIASymphony robot (Qiagen, Hilden, Germany) together with the QIAamp DNA Blood Midi Kit (Qiagen, Hilden Germany).

### Genome-wide genotyping

Genomic DNA from each dog was genotyped using the Illumina 170 K CanineHD BeadChip based on the CanFam3.1 (Illumina, San Diego, CA, USA)^47^. Genotyping of samples was performed at the Centre National de Genotypage (France) and NeoGen Genomics genotyping platform (NeoGen Genomics, Lincoln, NE, USA).

### Data filtering and visualization of population structure

Data filtering, sex confirmation and visualisation of population structure was performed using the software PLINK v1.90 ^33^. SNPs with a minor allele frequency (MAF) of less than 5% and SNPs which failed to be genotyped in more than 5% of samples were removed (–maf 0.05, –geno 0.05). Individuals with more than 5% genotyping data missing were removed (–mind 0.05). A sex check was performed to assure that the registered sex overlapped with the genotype predicted sex. A MDS plot was generated, and the first two dimensions (C1-C2) were plotted against each other to visualize population structure.

### GWAS analysis and visualization

A basic association test was performed with each phenotype using PLINK v1.90^33^. To account for cryptic relatedness and population structure, a univariate mixed model GWAS analysis was performed using the Genome-wide Efficient Mixed Model Association (GEMMA) software^48^. The software was used with a centered genotype matrix and Wald test statistics for statistical analysis. QQ-plots and Manhattan plots were generated in RStudio 2022.07.2 using the software package Fastman ^49,50^. The significance threshold for each association was determined using an LD corrected Bonferroni significance threshold based on the number of SNPs that were not in near-complete LD as determined by evaluating SNPs within 100 kb windows with an R^2^ > 0.9 as calculated by PLINK v1.90 (–indep 100 10 10)^51^. The proportion of phenotypic variance explained by the genotyping data (Chip heritability) was calculated for each linear mixed model analysis using GEMMA^48^.

### Additional statistical testing

Statistical testing was performed evaluating differences between phenotypes grouped by associated risk genotype. First, the dependent variables were tested to evaluate if they followed a normal distribution by a D’Agostino-Pearson test. For variables that followed a normal distribution, a one-way ANOVA analysis was performed to evaluate if there were significant differences within the dataset. This was followed by a Tukey’s multiple comparison test to evaluate the difference between individual genotypes, adjusting p-values for multiple testing. The EBV_HD_ was not normally distributed in its native or log transformed format. Hence, differences in EBV_HD_ between genotype groups were tested using a non-parametric Kruskal–Wallis test followed by a Dunn’s multiple comparison test, adjusting p-values for multiple testing. Linear regression analysis was used to evaluate any correlation between age and body weight. Graphical visualisation and analysis of the statistical tests was performed in Prism v. 10.0.2.

### Human GWAS comparison

The NHGRI-EBI GWAS catalog was used to evaluate if genes in the associated loci had been implicated in human studies investigating similar phenotypes^40^.

### Ethical approval

All blood samples included in this study were collected with owners informed written consent. Samples were collected in agreement with relevant guidelines and regulations. Ethical approval was granted by the regional animal ethics committee Uppsala ethics committee on animal experiments (Uppsala djurförsöksetiska nämnd) reference numbers: Dnr C12/15, D318/9, C139/9. Methods are reported in accordance with the ARRIVE guidelines.

### Supplementary Information

Supplementary Information 1.

Supplementary Information 2.

Supplementary Information 3.

Supplementary Information 4.

Supplementary Information 5.

## Data Availability

Phenotypic and genotyping data can be downloaded at SciLifeLab 10.17044/scilifelab.25334122.
