# Supplementary material for: Genome wide association study in Swedish Labrador retrievers identifies genetic loci associated with hip dysplasia and body weight
Source: Sci Rep. 2024 Mar 13;14:6090. doi: 10.1038/s41598-024-56060-y (PMC10937653; doi:10.1038/s41598-024-56060-y)
Supplement: Supplementary file 1 — Supplementary Information 1. [file 41598_2024_56060_MOESM1_ESM.pdf]

ID	ID	sex	HD	color	HDgrade	Weight	EBVHD
1	1	2	1	B	A	20	105.103
2	2	2	1	B	A	21	105.057
3	3	2	1	Y	A	22	109.037
4	4	2	1	B	A	22	107.549
5	5	2	1	B	A	22	112.08
6	6	2	1	B	A	22	101.828
7	7	2	1	B	A	23	99.238
8	8	2	1	B	A	23	106.592
9	9	2	1	B	A	23.4	100.14
10	10	2	1	B	A	24	105.63
11	11	2	1	B	A	24	103.822
12	12	2	1	B	A	24.7	109.267
13	13	2	1	B	A	25	107.705
14	14	2	1	B	A	25	111.99
15	15	2	1	B	A	25	109.346
16	16	2	1	Y	A	25.8	107.926
17	17	2	1	B	A	26	110.645
18	18	2	1	B	A	26.7	111.973
19	19	2	1	B	A	27	101.841
20	20	2	1	B	A	27	105.196
21	21	2	1	B	A	27	105.506
22	22	2	1	B	A	27	105.683
23	23	2	1	BR	A	27.3	98.485
24	24	2	1	B	A	28	103.086
25	25	2	1	Y	A	28	92.388
26	26	2	1	BR	A	28.3	108.679
27	27	2	1	BR	A	29	100.361
28	28	2	1	B	A	29	105.315
29	29	2	1	B	A	29	NA
30	30	2	1	B	A	29	103.168
31	31	2	1	B	A	29.8	108.287
32	32	2	1	B	A	30	105.804
33	33	2	1	Y	A	30	103.748
34	34	2	1	B	A	30	102.921
35	35	2	1	Y	A	30	99.079
36	36	2	1	B	A	30.5	97.634
37	37	2	1	BR	A	30.5	103.27
38	38	2	1	B	A	30.5	98.725
39	39	2	1	Y	A	30.6	105.755
40	40	2	1	B	A	31	104.055
41	41	2	1	B	A	32	104.022
42	42	2	1	Y	A	34	101.145
43	43	1	1	Y	A	34.5	NA
44	44	2	1	B	A	36	100.879
45	45	2	1	Y	A	38.2	104.467
46	46	2	1	B	A	NA	103.767
47	47	2	1	B	U	NA	94.056
48	48	2	1	Y	U	NA	99.563
49	49	2	1	B	A	NA	104.711
50	50	2	1	B	A	NA	97.742
51	51	2	1	B	U	NA	108.766
52	52	2	1	B	A	NA	112.63
53	53	2	1	B	A	NA	106.19

54	54	2	1	B	A	NA	106.861
55	55	2	1	B	A	NA	92.714
56	56	2	1	B	A	NA	103.958
57	57	2	1	B	A	NA	90.768
58	58	2	1	Y	A	NA	86.487
59	59	2	1	Y	A	NA	93.913
60	60	2	1	Y	A	NA	108.412
61	61	2	1	B	U	NA	91.459
62	62	2	1	Y	A	NA	106.011
63	63	2	1	B	A	NA	105.847
64	64	2	1	B	A	NA	103.081
65	65	2	1	B	A	NA	95.838
66	66	2	1	B	A	NA	84.46
67	67	2	1	B	A	NA	100.44
68	68	2	1	B	A	NA	101.162
69	69	2	1	B	A	NA	93.776
70	70	2	1	BR	A	NA	98.178
71	71	2	1	B	A	NA	94.65
72	72	2	1	B	A	NA	108.569
73	73	2	1	B	A	NA	92.813
74	74	2	1	B	A	NA	101.169
75	75	2	1	B	A	NA	108.003
76	76	2	1	Y	A	NA	109.326
77	77	2	1	B	A	NA	78.571
78	78	2	1	B	A	NA	103.701
79	79	2	1	B	A	NA	103.95
80	80	2	1	B	A	NA	107.62
81	81	2	1	BR	A	NA	103.61
82	82	2	1	B	A	NA	103.011
83	83	2	1	Y	A	NA	106.645
84	84	2	1	BR	A	NA	95.9
85	85	2	1	B	A	NA	102.829
86	86	2	1	BR	A	NA	107.187
87	87	2	1	B	A	NA	98.92
88	88	2	1	B	A	NA	104.507
89	89	2	1	B	A	NA	96.003
90	90	2	1	B	A	NA	102.758
91	91	2	1	B	A	NA	107.698
92	92	2	1	B	A	NA	103.992
93	93	2	1	B	A	NA	105.058
94	94	2	1	BR	A	NA	94.314
95	95	2	1	B	A	NA	109.634
96	96	2	1	BR	A	NA	105.356
97	97	2	1	BR	A	NA	102.634
98	98	2	1	BR	A	NA	103.921
99	99	2	1	B	A	NA	103.975
100	100	2	1	B	A	NA	103.645
101	101	2	1	Y	A	NA	101.166
102	102	2	1	BR	A	NA	103.054
103	103	2	1	B	A	NA	102.369
104	104	2	1	B	A	NA	104.141
105	105	2	1	B	A	NA	105.406
106	106	2	1	BR	A	NA	99.179
107	107	2	1	Y	A	NA	104.068

108	108	2	1	B	A	NA	104.903
109	109	2	2	BR	B	25	89.624
110	110	2	2	Y	B	25	97.376
111	111	2	2	Y	B	26	87.534
112	112	2	2	B	B	27	84.387
113	113	2	2	B	B	27	86.58
114	114	2	2	B	B	28.4	94.468
115	115	2	2	Y	B	29.5	89.99
116	116	2	2	Y	B	30	85.287
117	117	2	2	B	B	30	87.94
118	118	2	2	B	B	30	99.767
119	119	2	2	B	B	32	87.883
120	120	2	2	BR	B	35.4	97.497
121	121	1	2	Y	B	40	NA
122	122	2	2	B	B	NA	91.134
123	123	2	2	B	B	NA	91.987
124	124	2	2	B	B	NA	94.066
125	125	2	2	Y	B	NA	87.566
126	126	2	2	B	B	NA	84.179
127	127	2	2	B	B	NA	96.209
128	128	2	2	B	B	NA	86.856
129	129	2	2	B	B	NA	94.634
130	130	2	2	B	B	NA	95.624
131	131	2	2	Y	B	NA	106.249
132	132	2	2	Y	B	NA	94.346
133	133	2	2	B	B	NA	95.523
134	134	2	2	B	B	NA	91.728
135	135	2	2.5	B	C	26	92.362
136	136	2	2.5	B	C	26	93.187
137	137	2	2.5	B	C	29	91.378
138	138	2	2.5	B	C	30	78.906
139	139	2	2.5	B	C	31.7	92.333
140	140	2	2.5	B	C	33	90.845
141	141	2	2.5	B	C	NA	88.575
142	142	2	2.5	B	C	NA	82.885
143	143	2	2.5	Y	C	NA	89.312
144	144	2	2.5	B	C	NA	90.14
145	145	2	2.5	B	C	NA	89.414
146	146	2	3	B	D	30	88.294
147	147	2	3	Y	D	32	80.213
148	148	2	3	Y	D	NA	84.922
149	149	2	3	BR	D	NA	81.462
150	150	2	3	B	D	NA	77.468
151	151	2	1	B	A	18	102.576
152	152	2	1	B	A	21.6	106.76
153	153	2	1	Y	A	24.5	98.466
154	154	2	1	B	A	25	102.576
155	155	2	1	B	A	27	102.959
156	156	2	1	B	A	27	104.467
157	157	2	1	B	A	28	110.537
158	158	2	1	B	A	31	110.332
159	159	2	1	B	A	32	99.914
160	160	1	1	BR	A	34	102.822
161	161	2	1	B	A	NA	105.341

162	162	2	1	B	A	NA	101.955
163	163	2	1	B	A	NA	92.203
164	164	2	1	B	A	NA	109.252
165	165	2	1	B	A	NA	110.015
166	166	2	1	B	A	NA	103.052
167	167	2	1	B	A	NA	94.096
168	168	2	1	B	A	NA	113.913
169	169	2	1	Y	A	NA	98.726
170	170	2	1	B	A	NA	110.684
171	171	2	1	B	A	NA	91.855
172	172	2	1	BR	A	NA	101.62
173	173	2	1	Y	A	NA	108.111
174	174	2	1	B	A	NA	110.422
175	175	2	1	B	A	NA	108.686
176	176	2	1	BR	A	NA	98.926
177	177	2	2	B	B	25.5	102.935
178	178	2	2	B	B	27.2	NA
179	179	2	2	BR	B	30	91.126
180	180	2	2	B	B	32	94.487
181	181	2	2	B	B	NA	95.504
182	182	2	2	B	B	NA	95.363
183	183	2	2	B	B	NA	89.618
184	184	2	2	B	B	NA	97.819
185	185	2	2	B	B	NA	96.559
186	186	2	2	B	B	NA	89.057
187	187	2	2	B	B	NA	97.897
188	188	2	2	B	B	NA	96.012
189	189	2	2.5	B	C	20	93.987
190	190	1	2.5	B	C	24.6	86.306
191	191	2	2.5	Y	C	27.5	98.76
192	192	2	2.5	Y	C	33.5	78.279
193	193	2	2.5	B	C	35	93.722
194	194	2	2.5	B	C	NA	85.155
195	195	2	2.5	B	C	NA	81.904
196	196	2	3	Y	D	26	69.782
197	197	2	3	Y	D	NA	84.039
198	198	2	3	BR	D	NA	83.861
199	199	2	3	B	M	NA	85.238
200	200	2	1	B	A	NA	104.444
201	201	2	2	Y	B	24	92.569
202	202	2	2	B	B	NA	82.276
203	203	2	2	B	B	NA	101.763
204	204	2	2	B	B	NA	88.942
205	205	2	2	B	B	NA	84.63
206	206	2	2.5	B	C	20	90.857
207	207	2	3.7	Y	E	34.4	71.874
208	208	2	3.7	B	E	NA	72.596
209	209	2	3.7	B	E	NA	69.757

Summary of phenotypic input data for the GWAS study. Note that 5 dogs where HD scored according to a predated scoring system. U=Normal hips translated as a categorical score A and a numerical score 1. and M=Moderate hip dysplasia translated as categorical

score D and a numerical score 3.
