# Supplementary material for: Genome wide association study in Swedish Labrador retrievers identifies genetic loci associated with hip dysplasia and body weight
Source: Sci Rep. 2024 Mar 13;14:6090. doi: 10.1038/s41598-024-56060-y (PMC10937653; doi:10.1038/s41598-024-56060-y)
Supplement: Supplementary file 2 — Supplementary Information 2. [file 41598_2024_56060_MOESM2_ESM.pdf]

SNPs associated with numerical HD score with p_wald < 10E-4. In green SNPs considered significantly associated based on the LD corrected significance threshold are highlighted.

chr	rs	ps	n_miss	allele1	allele0	af	beta	se	logl_H1	l_remle	p_wald
24	BICF2G630501155	25406999	0	G	A	0.165	4.15E-01	7.95E-02	-2.04E+02	1.00E-05	4.35E-07
24	BICF2G630501140	25417878	0	C	T	0.165	4.15E-01	7.95E-02	-2.04E+02	1.00E-05	4.35E-07
24	BICF2G630501113	25465028	0	T	G	0.165	4.15E-01	7.95E-02	-2.04E+02	1.00E-05	4.35E-07
24	BICF2G630501077	25517368	1	C	T	0.163	4.07E-01	8.00E-02	-2.04E+02	1.00E-05	8.02E-07
24	BICF2G630501177	25370385	0	A	G	0.177	3.82E-01	7.80E-02	-2.05E+02	1.00E-05	1.98E-06
24	BICF2G630501310	25171633	0	G	A	0.191	3.40E-01	7.46E-02	-2.06E+02	1.00E-05	8.57E-06
24	BICF2P677598	28071200	0	A	G	0.179	3.94E-01	8.77E-02	-2.05E+02	5.58E-01	1.18E-05
24	BICF2P1470166	24945587	0	A	T	0.175	3.59E-01	8.00E-02	-2.07E+02	1.00E-05	1.21E-05
24	BICF2P1231914	25917665	1	C	T	0.204	3.28E-01	7.34E-02	-2.07E+02	1.00E-05	1.29E-05
22	BICF2G63092169	60292790	0	A	G	0.419	3.30E-01	7.45E-02	-2.05E+02	1.64E+00	1.51E-05
24	BICF2P823536	27934616	0	C	T	0.148	4.17E-01	9.47E-02	-2.06E+02	2.60E-01	1.67E-05
36	TIGRP2P414879	26483200	0	G	A	0.395	3.06E-01	7.15E-02	-2.06E+02	2.90E-01	2.82E-05
29	BICF2P1301971	37583170	0	G	A	0.079	5.29E-01	1.27E-01	-2.06E+02	1.44E+00	4.30E-05
24	BICF2G630500919	26552009	0	A	G	0.069	4.93E-01	1.19E-01	-2.08E+02	1.00E-05	4.95E-05
24	BICF2G630500769	26713056	0	A	G	0.069	4.93E-01	1.19E-01	-2.08E+02	1.00E-05	4.95E-05
24	BICF2G630500736	26759853	0	G	A	0.069	4.93E-01	1.19E-01	-2.08E+02	1.00E-05	4.95E-05
24	BICF2G630500846	26656491	0	A	T	0.079	4.70E-01	1.14E-01	-2.08E+02	1.00E-05	5.21E-05
29	BICF2P585594	37787989	0	G	A	0.108	4.39E-01	1.09E-01	-2.06E+02	8.84E-01	7.43E-05
24	BICF2S23030320	38827337	0	T	C	0.06	5.99E-01	1.48E-01	-2.06E+02	1.49E+00	7.56E-05
12	BICF2S23624850	53825022	0	A	G	0.333	2.67E-01	6.69E-02	-2.07E+02	8.99E-01	9.12E-05

SNPs associated body weigh with p_wald < 10E-4. In green SNPs considered significantly associated based on the LD corrected significance threshold are highlighted.

chr	rs	ps	n_miss	allele1	allele0	af	beta	se	logl_H1	l_remle	p_wald
10	BICF2S23722746	48367568	0	C	T	0.324	-2.95E+00	5.39E-01	-2.10E+02	1.42E+00	4.48E-07
31	BICF2P1132508	1938609	0	T	C	0.112	3.88E+00	7.66E-01	-2.10E+02	1.67E+01	2.48E-06
31	BICF2P271586	1948675	0	G	A	0.112	3.88E+00	7.66E-01	-2.10E+02	1.67E+01	2.48E-06
31	BICF2P1027221	2112899	0	T	C	0.112	3.88E+00	7.66E-01	-2.10E+02	1.67E+01	2.48E-06
31	BICF2P891982	2120727	0	C	T	0.112	3.88E+00	7.66E-01	-2.10E+02	1.67E+01	2.48E-06
31	BICF2P405398	2385304	0	C	T	0.106	3.72E+00	8.17E-01	-2.12E+02	8.92E+00	1.83E-05
31	BICF2P344565	2388037	0	T	C	0.106	3.72E+00	8.17E-01	-2.12E+02	8.92E+00	1.83E-05
10	BICF2P432142	48590235	0	G	A	0.247	-2.88E+00	6.39E-01	-2.14E+02	1.21E+00	2.14E-05
3	BICF2S23243416	25894843	0	C	T	0.229	-2.79E+00	6.22E-01	-2.13E+02	3.52E+00	2.31E-05
7	BICF2G630551463	11639564	0	G	T	0.335	-2.46E+00	5.49E-01	-2.12E+02	2.43E+01	2.42E-05
20	TIGRP2P278627	57621328	0	A	G	0.106	3.68E+00	8.23E-01	-2.12E+02	1.00E+05	2.42E-05
22	BICF2G630318243	11645141	0	C	T	0.494	-2.23E+00	5.03E-01	-2.12E+02	1.00E+05	2.69E-05
31	BICF2G630727135	2210422	0	A	C	0.124	3.35E+00	7.59E-01	-2.12E+02	3.09E+01	3.00E-05
10	BICF2P403249	48563527	0	G	A	0.229	-2.80E+00	6.49E-01	-2.15E+02	1.27E+00	4.44E-05
22	BICF2S23626812	19305475	0	A	G	0.288	-2.54E+00	5.91E-01	-2.12E+02	3.59E+01	4.74E-05
3	BICF2P1106056	51117598	0	A	G	0.135	-3.45E+00	8.26E-01	-2.13E+02	4.34E+00	7.16E-05
7	BICF2G630551534	11763582	0	C	A	0.418	-2.31E+00	5.54E-01	-2.13E+02	8.35E+01	7.41E-05
7	BICF2G630551535	11764258	0	C	T	0.418	-2.31E+00	5.54E-01	-2.13E+02	8.35E+01	7.41E-05
17	BICF2P1041331	63173812	0	A	G	0.347	-2.06E+00	5.02E-01	-2.13E+02	3.24E+01	9.33E-05
10	BICF2G630479089	21256164	0	C	T	0.265	-2.45E+00	5.97E-01	-2.13E+02	8.55E+00	9.49E-05
31	BICF2G630741948	34355737	0	A	G	0.188	-2.62E+00	6.40E-01	-2.13E+02	3.96E+01	9.60E-05
