# Supplementary material for: Genome wide association study in Swedish Labrador retrievers identifies genetic loci associated with hip dysplasia and body weight
Source: Sci Rep. 2024 Mar 13;14:6090. doi: 10.1038/s41598-024-56060-y (PMC10937653; doi:10.1038/s41598-024-56060-y)
Supplement: Supplementary file 3 — Supplementary Information 3. [file 41598_2024_56060_MOESM3_ESM.pdf]

Supplementary figures S1-S5

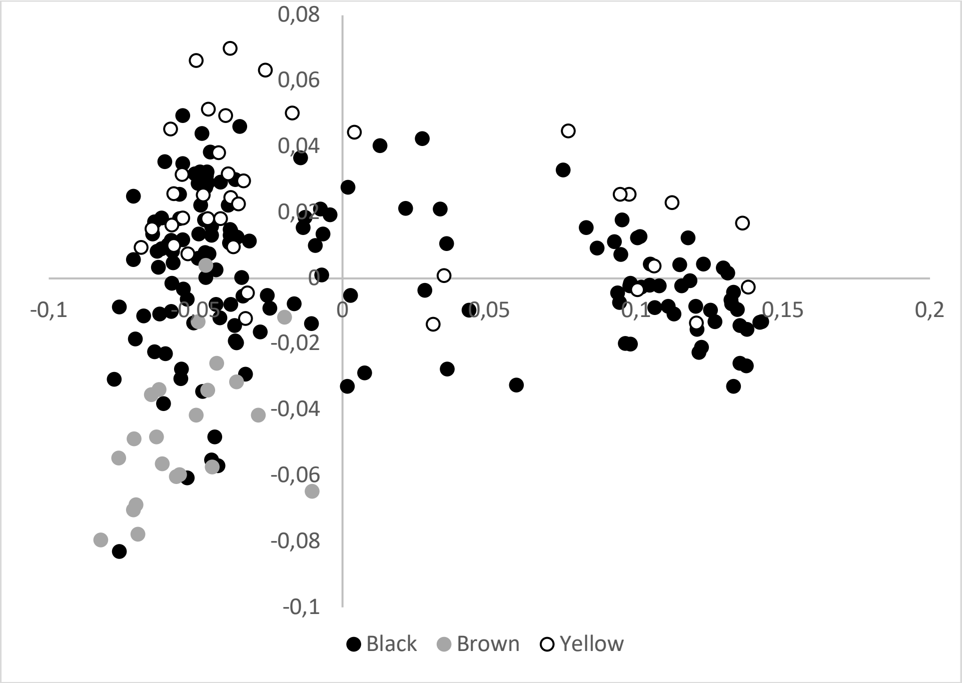

Supplementary figure S1: Multidimensional scaling plot depicting the two first components C1 and C2 reflecting the genomic distance between each individual in the dataset. The color of each individual is reflected in the datapoints. Some population structure is observed within the population. Though the black and yellow dogs appear equally distributed across the plot, the brown dogs appear to cluster separately.

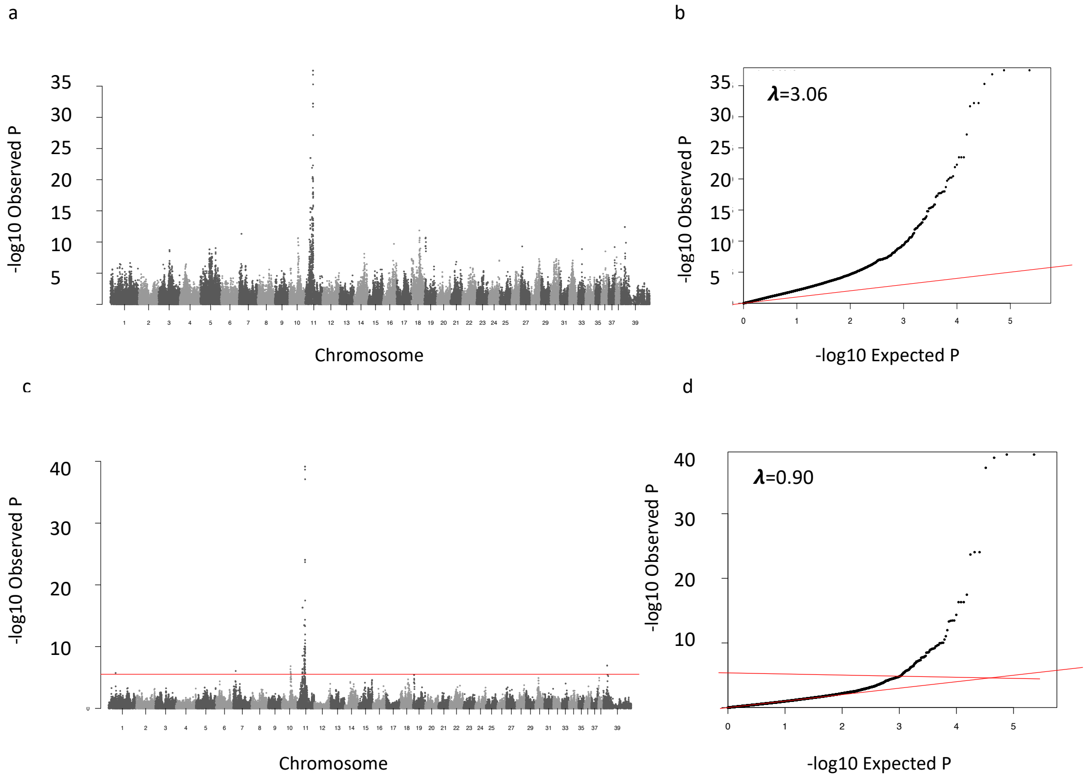

Supplementary figure S2: GWAS using 148 black versus 22 brown coat colors as phenotype. A) Manhattan plot illustrating results from basic association test performed using Plink v1.9. B) QQ-plot illustrating distribution of p-values from basic association test performed using Plink v1.9. C) Manhattan plot illustrating results from linear mixed model association test performed using GEMMA 0.98.1. D) QQ-plot illustrating distribution of p-values from basic association test performed using GEMMA 0.98.1. Significance threshold illustrated in C) and D) is Bonferroni corrected based on number of independent SNPs evaluated in the analysis (--indep 100 10 10).

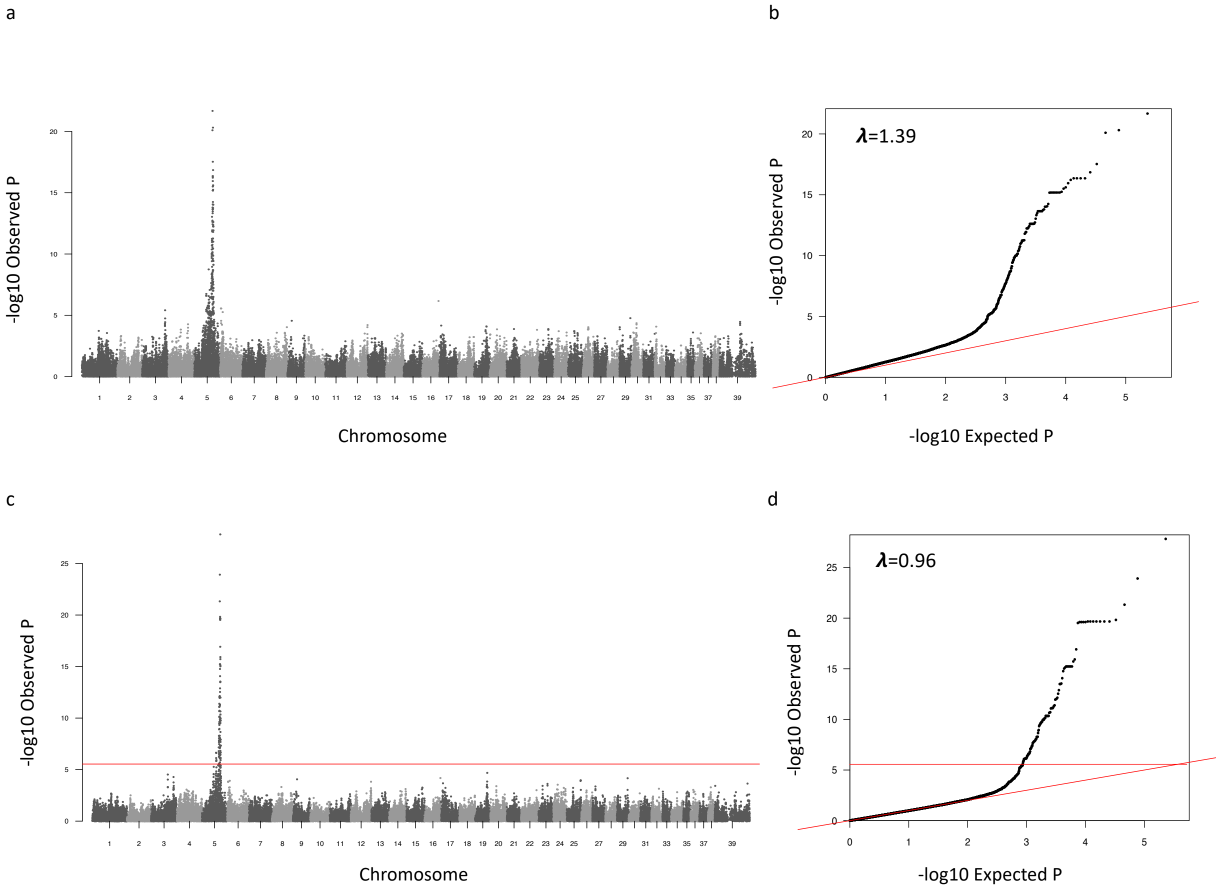

Supplementary figure S3: GWAS using 148 black versus 38 yellow coat colors as phenotype. A) Manhattan plot illustrating results from basic association test performed using Plink v1.9. B) QQ-plot illustrating distribution of p-values from basic association test performed using Plink v1.9. C) Manhattan plot illustrating results from linear mixed model association test performed using GEMMA 0.98.1. D) QQ-plot illustrating distribution of p-values from basic association test performed using GEMMA 0.98.1. Significance threshold illustrated in C) and D) is Bonferroni corrected based on number of independent SNPs evaluated in the analysis (--indep 100 10 10).

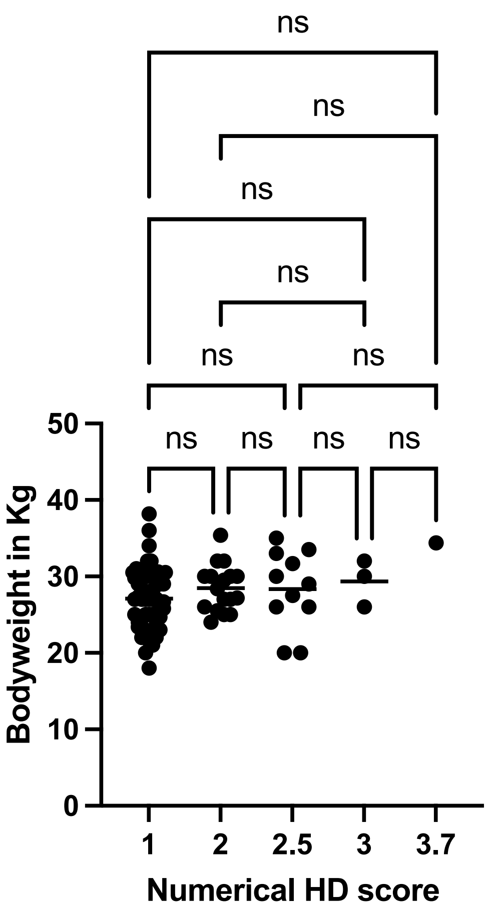

Supplementary figure S4: Scatter dot plot showing the weight in relation to the different numerical HD score groups. Horizontal line indicates mean weight in group. One-way ANOVA test di not find any significant difference between groups p=0.26. ns=not significant p>0.05.

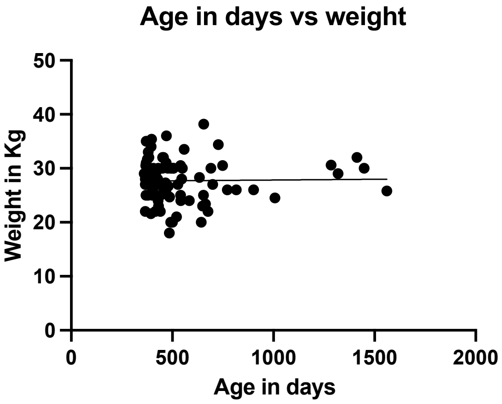

Supplementary figure S5: Simple linear regression comparing age in days with weight in kg. No significant correlation was found between age and weight R^2=0.0003 and p=0.87.

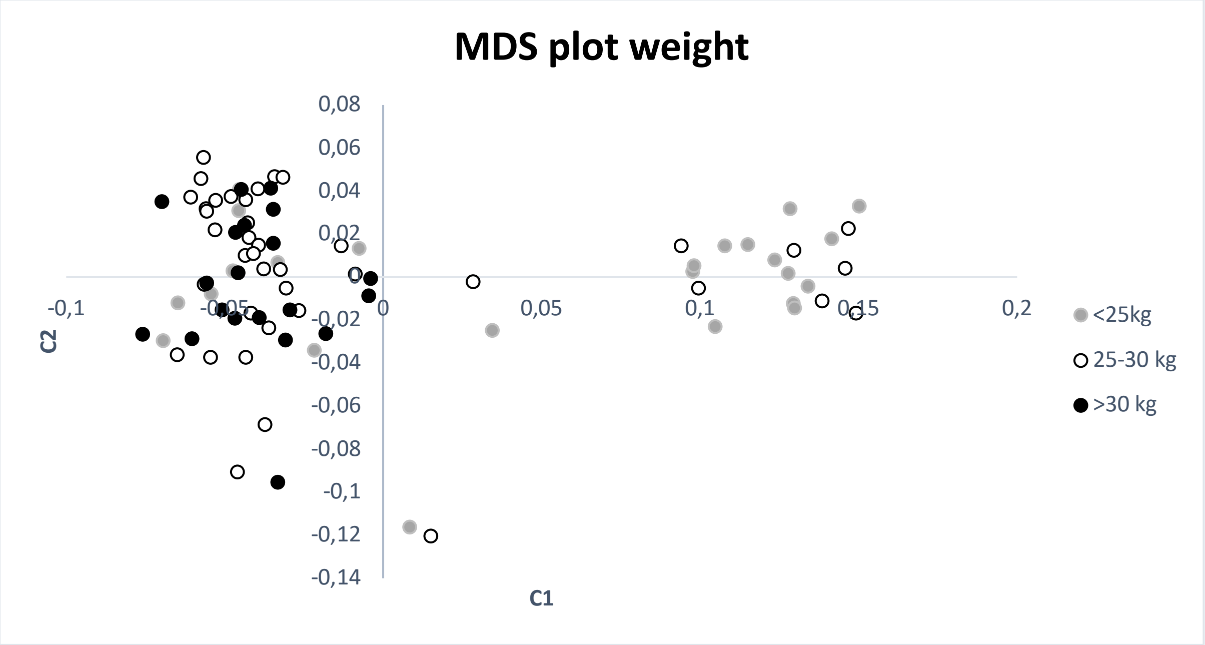

Supplementary figure S6: Multidimensional scaling plot showing the genetic distance between the 85 genotyped Labrador retrievers in a two-dimensional space. Each datapoint represents an individual dog and the color of the datapoint reflects the body weight. ● represents individuals weighing less than 25kg ○ represents individuals weighing between 25 and 30kg. ● represents individuals weighing more than 30 kg.
