# Supplementary material for: Genome wide association study in Swedish Labrador retrievers identifies genetic loci associated with hip dysplasia and body weight
Source: Sci Rep. 2024 Mar 13;14:6090. doi: 10.1038/s41598-024-56060-y (PMC10937653; doi:10.1038/s41598-024-56060-y)
Supplement: Supplementary file 4 — Supplementary Information 4. [file 41598_2024_56060_MOESM4_ESM.pdf]

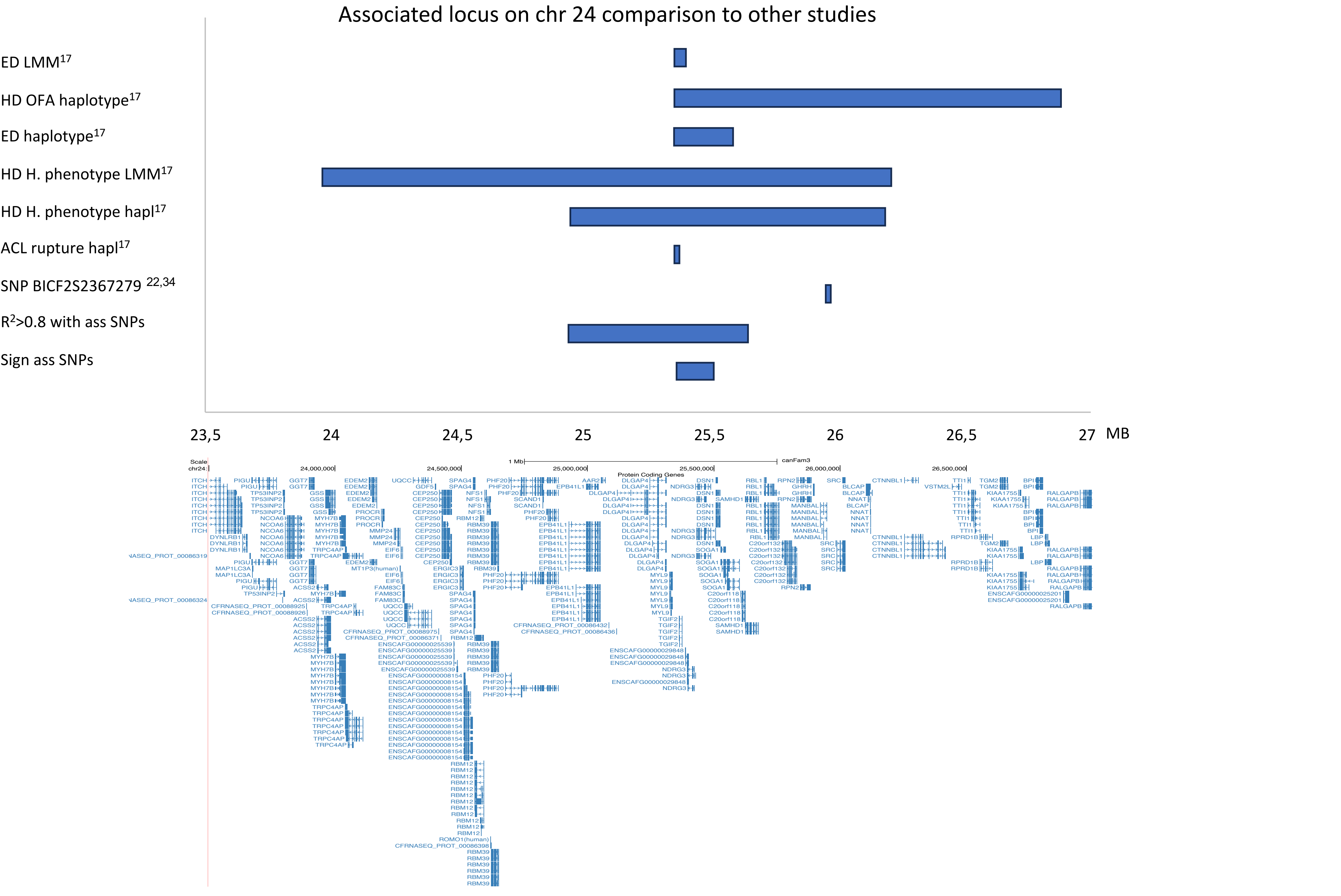
Associated locus on chr 24 comparison to other studies
ED LMM17
HD OFA haplotype17
ED haplotype17
HD H. phenotype LMM17
HD H. phenotype hapl17
ACL rupture hapl17
SNP BICF2S2367279 22,34
R2>0.8 with ass SNPs
Sign ass SNPs
23,5
24
24,5
25
25,5
26
26,5
27
MB
Scale
chr24:
1 Mb
canFam3
24,000,000
24,500,000
25,000,000
25,500,000
26,000,000
26,500,000
Protein Coding Genes

Supplementary figure S7: Figure showing the overlap between the locus on chr 24 associated with HD in the current study and comparison to other overlapping loci described by Fels & Distl 2014, Mikkola et al. 2021 and Binversie et al 2022[17, 22, 32].

Labels: ED LMM = Overlapping locus on chr 24 associated with elbow dysplasia in a linear mixed model association analysis[17]. HD OFA haplotype: = Overlapping locus on chr 24 associated with hip dysplasia based on haplotype association[17]. ED haplotype = Overlapping locus on chr 24 associated with elbow dysplasia based on haplotype association[17]. HD H. phenotype LMM = Overlapping locus on chr 24 associated with hip dysplasia in a linear mixed model association analysis based on hospital record estimated phenotypes[17]. HD H. phenotype hapl= ACL rupture hapl. = [17] SNP BICF2S2367279 = Single SNP identified to be associated with hip dysplasia in German shepherds and later shown to be associated with hip dysplasia in Labrador retrievers and Samoyeds [22,34]. R^2>0.8 with ass SNPs = Area covering SNPs which are in high LD with the most associated SNPs identified in the current study. Sign ass SNPs = Location of the significantly associated SNPs identified in the current study.

The UCSC browser track overlapping the area (chr24:24.5-27.0 MB)for CanFam3.1 is shown below to visualize gene in transcripts.
