# Supplementary material for: Genome wide association study in Swedish Labrador retrievers identifies genetic loci associated with hip dysplasia and body weight
Source: Sci Rep. 2024 Mar 13;14:6090. doi: 10.1038/s41598-024-56060-y (PMC10937653; doi:10.1038/s41598-024-56060-y)
Supplement: Supplementary file 5 — Supplementary Information 5. [file 41598_2024_56060_MOESM5_ESM.docx]

**Supplementary figures and files**

Supplementary figure S1- S5

Supplementary file S1. Summary of individual IDs and phenotypes for the genotyped Labrador retrievers included in the study.

Supplementary file S2. Summary of SNPs reaching nominal significance as based on deviation from the expected p-value at p<10^-4^.
